# Metabolite Profiles, Bioactivity, and HPLC Fingerprint of Different Varieties of *Eucommia ulmoides* Oliv.: Towards the Utilization of Medicinal and Commercial Chinese Endemic Tree

**DOI:** 10.3390/molecules23081898

**Published:** 2018-07-30

**Authors:** Dong Wu, Danmeng Yu, Yujia Zhang, Juane Dong, Dengwu Li, Dongmei Wang

**Affiliations:** 1 College of Forestry, Northwest A&F University, Yangling 712100, Shaanxi, China; dongwu0925@163.com (D.W.); diamondyu25@163.com (D.Y.); 15591822108@163.com (Y.Z.); dengwuli@163.com (D.L.); 2 College of Life Sciences, Northwest A&F University, Yangling 712100, Shaanxi, China; dzsys@nwsuaf.edu.cn

**Keywords:** *Eucommia ulmoides* Oliv., different varieties, metabolite profiles, bioactivity, HPLC fingerprint, chemometrics analysis

## Abstract

*Eucommia ulmoides* Oliv. is widely regarded in China as a precious medicinal and commercial endemic tree. Due to cross-breeding or natural variation of *E. ulmoides*, the metabolite composition may vary significantly, making control of the medical quality difficult. In order to improve the rational development and utilization, the quality of seven varieties of *E. ulmoides* were evaluated based on metabolite profiles (total phenolic, total flavonoid, gutta-percha, aucubin, geniposidic acid, chlorogenic acid, geniposide, pinoresinol diglucoside, rutin, hyperoside, and astragalin), bioactivities (in vitro, in vivo antioxidant activities, and antibacterial activities) and HPLC fingerprint combined with chemometrics analysis. On this basis, the differences of medicinal parts (leaf and bark) were further carried out. For the traditional use of bark, Purple-leaf *E. ulmoides* was the most suitable. For the use of leaf, Qinzhong 1 and Purple-leaf *E. ulmoides* were appropriate. HPLC fingerprint analysis showed that significant differences in metabolite profiles exist among seven varieties of *E. ulmoides*. Combined with chemometrics analysis, seven varieties of *E. ulmoides* were divided into three groups from the use of leaf and bark. The analysis not only evaluated quality of seven varieties of *E. ulmoides*, but also could distinguish different varieties and different regions of origin. The results can provide theoretical basis for *E. ulmoides* resources utilization and cultivation of fine varieties.

## 1. Introduction

*Eucommia ulmoides* Oliv., also known as Du Zhong, is a deciduous and dioecious tree belonging to monotypic genus *Eucommia*. The extracts of the aerial parts of this plant have been widely used in famous botanical tonics medicine of health care for more than 2000 years as an endemic Chinese herb [1]. *E. ulmoides* has been traditionally used in various indigenous systems of medicines. *E. ulmoides* is indigenous to China and is widely distributed in Henan, Sichuan, and other places, and the wild distribution center is located in western China [2,3]. Besides medical benefits, *E. ulmoides* has high values in the development of commercial products; the flowers have been prepared as a natural health-care tea [4]. *E. ulmoides* seed oil can be used for food and nutritional vegetable oil. Gutta-percha is available from the specific tissues or organs of *E. ulmoides*, and it is used extensively in electrical insulation and as filling material in dentistry [5]. Many researches showed that all parts of *E. ulmoides* have huge potential for exploitation and can be utilized in the industrial scale [6].

Studies have shown that *E. ulmoides* exhibits rich pharmacological activities, including antioxidant [7,8], antimicrobial, anti-inflammatory [9], antihypertensive [10,11], and antineoplastic [12]. The *E. ulmoides* leaf showed a stronger antioxidant activity compared with the bark, flower, and fruit, and the leaf illustrated a potential application as a commercial antioxidant [13]. Previous studies have shown that ethyl acetate extract of *E. ulmoides* flowers had good antibacterial activities against *Staphylococcus aureus*, *Bacillus anthracis*, *Bacillus subtilis*, and *Escherichia coli* [14]. The effective components of *E. ulmoides* include phenolics, flavonoids (such as quercetin and rutin), iridoids (such as geniposide, aucubin, and geniposidic acid), phenylpropanoids (such as chlorogenic acid), lignans (pinoresinol diglucoside), sterols, terpenoids, and polysaccharides [15,16]. Flavonoids are strong antioxidants that can inhibit carcinogenesis [17,18]. Geniposidic acid exhibited the effect against aging and inflammation and promoted collagen synthesis [19,20]. Aucubin is well known to exhibit parasympathetic stimulation and antimicrobial activity [21]. Chlorogenic acid, a major bioactive compound found in the *E. ulmoides*, has several pharmacological activities, including antilipid peroxidation, liver protection, antimicrobial and antiviral activity, and spasmolysis [22]. Pinoresinol diglucosides belonging to lignans with various pharmacological functions have been identified as the main antihypertensive compounds in the bark of *E. ulmoides* [23].

*E. ulmoides* is widely applied in China and abroad owing to its high medicinal and commercial value. Due to cross-breeding or the natural variation of *E. ulmoides*, different types of internal variation were produced, such as Huazhong 1–9, Qinzhong 1–4, and purple-leaf *E. ulmoides*. Because the quality is determined by its secondary metabolites, the variation resulted in metabolite compositions that varied significantly, and made control of the medical quality difficult [24,25]. All parts of the *E. ulmoides* plant nearly contain the same compounds, but due to different production locations or varieties, secondary metabolites that are contained in medicinal parts may be different in their content and proportion of constituents [26,27]. Therefore, it is of high significance to identify the quality of *E. ulmoides* and to select excellent varieties from the aspects of metabolite profiles and bioactivities.

In this work, the quality of seven varieties of *E. ulmoides* were evaluated for the first time based on metabolite profiles, bioactivity, and their HPLC fingerprint combined with chemometrics analysis. The results can provide theoretical basis for *E. ulmoides* resource utilization and germplasm resources evaluation.

## 2. Results and Discussion

Seven varieties of *E. ulmoides* were collected in August for the study. S1–S7 were used to represent 7 varieties of *E. ulmoides. E. ulmoides* (S1) is a conventional variety of *Eucommia*, it was collected from Northwest A & F University, Yangling, Shaanxi Province, China. Qinzhong 1–4 (S2–S5) are new excellent varieties with a high content of gutta-percha and high medicinal activity; they were collected from Northwest A&F University, Yangling, Shaanxi Province, China. Purple-leaf *E. ulmoides* (S6) and Short-branch and dense leaf *E. ulmoides* (S7) are new ornamental and medicinal varieties of *Eucommia*, they were collected from Northwest A&F University Ankang North Subtropical Economic Forest Fruit Tree Experiment Demonstration Station, Shaanxi Province, China.

## 3. Contents of Total Phenolic, Total Flavonoid, and Gutta-Percha

Seven varieties of *E. ulmoides* were rich in total phenolic, total flavonoid, and gutta-percha, the contents were presented in Table 1. The total phenolic contents ranged from 9.2 ± 1.6 (leaf of Qinzhong 3, S4) to 95.5 ± 3.7 mmol equiv. gallic acid (GAE)/100 g (leaf of Purple-leaf *E. ulmoides*, S6) and the contents of the leaves were generally higher than the bark. The contents of total flavonoid were 44.2 ± 4.1–550.4 ± 4.4 mmol equiv. quercetin (QUE)/100 g. For Purple-leaf *E. ulmoides* (S6) and *E. ulmoides* (S1), the total flavonoid contents of the leaves were generally higher than the bark, but the contents of the other five varieties were the opposite. Qinzhong 1 (S2) contained the highest total flavonoid content of 550.4 ± 4.4 mmol equiv. QUE/100 g. For contents of gutta-percha of the seven varieties of *E. ulmoides*, the bark (2.56% ± 0.13 to 5.46% ± 0.12) was generally higher than leaf (1.09% ± 0.12 to 3.33% ± 0.08). The highest flavonoid content was observed in the Purple-leaf *E. ulmoides* (S6) in the bark (5.46% ± 0.12). Qinzhong 3 (S4) had the lowest content (1.09% ± 0.12).

The results showed that the total phenolic, total flavonoid, and gutta-percha contents of different varieties of *E. ulmoides* were significantly different. The significant difference was also found in the samples of different parts. Since the samples were collected from different varieties, these differences may be due to diversity in varieties during the organic and developmental stage of biology.

## 4. HPLC Analysis of Eight Compounds of Different *E. ulmoides* Varieties

### 4.1. Validation of the Method

**Figure 1:**
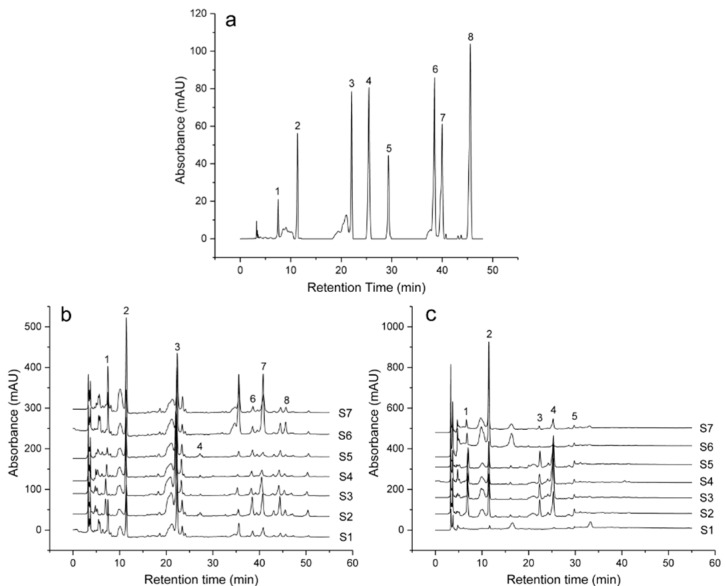
HPLC analysis of the eight mixed standards (**a**), and the HPLC fingerprinting profiles for leaf (**b**) and bark (**c**) of different *E. ulmoides* varieties. (**a**) The peaks 1–8 and the wavelength were aucubin (208 nm), geniposidic acid (238 nm), chlorogenic acid (238 nm), geniposide (238 nm), pinoresinol diglucoside (238 nm), rutin (254 nm), hyperoside (254 nm), and astragalin (254 nm), respectively.

Under the optimal conditions, eight well-separated compounds were detected in the leaf and bark. The precision of the analytical method was determined by assaying six replicates of the compounds, the relative standard deviation (RSD) values of the peak area were estimated to be 0.14–2.51% (*n* = 6). A recovery experiment (Table S1) was performed to confirm the accuracy of the method by mixing quantified samples with standard compounds in the appropriate amount. The average percentages of recovery of the eight compounds were at different levels and ranged from 96.36% ± 0.98 to 106.43% ± 1.28 and RSD varied from 1.09% to 1.87% (*n* = 6). The repeatability of the method was detected by extracting one sample for six times, while the area of the peaks were recorded, the RSD of the area varied from 1.01 to 1.91% (*n* = 6). The stability of the eight compounds in the sample solution was evaluated by determining their relative peak areas after storage at room temperature for 2, 4, 8, 16, and 24 h, respectively. All results were summarized in Table 2 and demonstrated that the conditions for the analysis were repeatable and accurate. The retention time of the eight mixed standards were showed in Figure 1a.

### 4.2. Contents of 8 Compounds of Different Varieties E. ulmoides

Chromatograms of leaf and bark were analyzed by the HPLC method. Based on the retention time of the sample peaks compared to those of the standards, peaks were identified as aucubin, geniposidic acid, chlorogenic acid, geniposide, pinoresinol diglucoside, rutin, hyperoside, and astragalin (Figure 1a).

The contents of the eight compounds were summarized in Table 3. The contents of the eight compounds of the leaf and bark showed Qinzhong 1 (S2) had the highest value of the eight compounds, and *E. ulmoides* (S1) had the lowest value. Qinzhong 1 (S2) had the highest content of chlorogenic acid (120.0 ± 0.7 mg/g), aucubin (368.6 ± 1.0 mg/g), rutin (15.1 ± 0.1 mg/g), and hyperoside (29.0 ± 0.3 mg/g). In addition, Purple-leaf *E. ulmoides* (S6) had the highest value of astragalin (5.9 ± 0.1 mg/g) and geniposidic acid (230.2 ± 1.6 mg/g). The results showed that rutin, hyperoside, and astragalin were only detected in leaf, and pinoresinol diglucoside was only detected in bark. For leaf, geniposide was detected only in Qinzhong 1–4 (S2, S3, S4 and S5). Among the seven varieties, the contents of these compounds were showed to be significantly different (*p* < 0.05).

## 5. Antioxidant Activity

### 5.1. In Vitro Antioxidant Activity (DPPH, ABTS, and FRAP Assays)

The results of in vitro antioxidant activity were listed in Table 4. In these assays, all extracts showed a notable radical scavenging activity in a dose-dependent manner within a certain range of DPPH (1,1-diphenyl-2-picrylhydrazyl), the capacity to neutralize the radical cation ABTS•+ (2,2′-azino-bis(3-ethylbenzothiazoline-6-sulfonic acid) and the reducing ability of FRAP (Ferric ion reducing antioxidant power) were significantly different (*p* < 0.05). The range of DPPH with the IC_50_ was 1.0 ± 0.7 μg/mL (leaf of Purple-leaf *E. ulmoides*, S6)–56.5 ± 0.4 μg/mL (bark of *E. ulmoides*, S1), the values of ABTS were from 902.8 ± 3.8 μmol equiv. Trolox/g (bark of Qinzhong 3, S4) to 7471.4 ± 2.7 μmol equiv. Trolox/g (leaf of Purple-leaf *E. ulmoides*, S6) and the values of FRAP were from 514.5 ± 2.1 μmol equiv. Trolox/g (bark of *E. ulmoides*, S1) to 6161.6 ± 3.0 μmol equiv. Trolox/g (leaf of Purple-leaf *E. ulmoides*, S6). Of the three antioxidant activity methods in vitro, the highest activity was obtained from Purple-leaf *E. ulmoides* (S6). The lowest was obtained from *E. ulmoides* (S1) with the method of DPPH and FRAP. For ABTS, the lowest activity was obtained from Qinzhong 3 (S4). The values of DPPH, ABTS, and FRAP showed the antioxidant activity of leaf was generally stronger than bark.

The three in vitro antioxidant activity methods (DPPH, ABTS, and FRAP) ranked the varieties of *E. ulmoides* differently. Because the three in vitro antioxidant activity methods were different in principle, the target of radicals and ions were distinct. The determination of antioxidant activity requires a combination of methods. Based on previous studies, we selected these three methods to determine antioxidant activity of the varieties of *E. ulmoides* [28,29]. Combined with these methods, we could screen the antioxidant activity of the different varieties, and Purple-leaf *E. ulmoides* was the benefit antioxidant activity variety.

### 5.2. In Vivo Antioxidant Activity (MTS Assay)

The MTS antioxidant activities of leaf and bark were showed in Table 4. All tested samples exerted a protective effect against the damage of H_2_O_2_. The results showed that the values of MTS were 1.3 ± 0.1 (bark of *E. ulmoides*, S1) and –2.5 ± 0.1 (leaf of Purple-leaf *E. ulmoides*, S6). Purple-leaf *E. ulmoides* (S6) possessed a higher protective effect against the damage of H_2_O_2_ with values of 2.5 ± 0.1. The values obtained from Qinzhong 1–4 (S2, S3, S4, and S5) varieties were not significantly different; however, the other varieties were significantly different (*p* < 0.05).

Based on antioxidant activity results, it is possible to infer that Purple-leaf *E. ulmoides* not only presented the highest free radical scavenge capacity and strongest reducing capacity, but also exerted a protective effect against the damage of H_2_O_2_. Generally, the antioxidant activities of leaf were markedly stronger than bark. Different samples showed significantly different antioxidant activities.

In this study, antioxidant activities were evaluated by four methods. The order of the antioxidant activities measured by these four methods were slightly different. Therefore, to fully understand the antioxidant activity of these varieties requires further analysis.

## 6. Antibacterial and Antifungal Activities

The seven varieties of *E. ulmoides* had been studied for the antibacterial and antifungal activities against nine bacteria and one fungus. Using the paper-disc agar diffusion method, the DIZ (diameter of the inhibition zones) (Table S2) of leaf and bark extracts were measured, and the most suitable strains were screened for MIC, MBC, and EC_50_. By this procedure, six bacteria (*E. coli*, *P. aeruginos*, *S. paratyphi*, *S. typhimurium*, *B. subtilis*, and *S. aurous*) were chosen for analysis of MIC and MBC (Table 5). Five bacteria and one fungus (*S. enteritidis*, *P. aeruginosa*, *S. paratyphi*, *S. typhimurium*, *S. aurous*, and *C. albicans*) were chosen for the analysis of EC_50_ (Table 6). The qualities of seven varieties of *E. ulmoides* were further evaluated by MIC, MBC, and EC_50_.

The MIC and MBC values for all tested bacterial strains ranged from 0.3125 to 10.00 mg/mL. In our study, the MIC and MBC values of leaf extracts showed stronger antibacterial activity. Extract of Purple-leaf *E. ulmoides* (S6) showed the strongest activity against the bacteria *S. paratyphi*, *S. aurous*, and *B. subtilis* with the value of 0.3125 mg/mL. Extract of *E. ulmoides* (S1) had a strong inhibitory effect against the bacteria *S. paratyphi*, *S. aurous*, *E. coli*, and *P. aeruginosa*. Extract of short-branch and dense leaf *E. ulmoides* (S7) had relatively strong bactericidal effect against the bacteria *B. subtilis*. For the result of EC_50_, seven varieties of *E. ulmoides* showed various degrees of inhibition against the bacteria and fungi tested. Extract of short-branch and dense leaf *E. ulmoides* (S7) exhibited the strongest inhibition for *P. aeruginosa*, *S. paratyphi*, and *S. aurous* with the EC_50_ values of 12.9 ± 0.3, 9.7 ± 0.1 and 10.6 ± 0.1 mg/mL, respectively. Extract of *E. ulmoides* (S1) showed the strongest inhibition activity for *S. typhimurium* and *C. albicans*, the EC_50_ values were 13.9 ± 0.4 and 13.2 ± 0.3 mg/mL, respectively. The results of our study showed significant differences for different varieties (*p* < 0.05).

The results of EC_50_ were slightly different for MIC and MBC. In conclusion, all seven varieties of *E. ulmoides* showed broad-spectrum antibacterial and antifungal activity. Samples of Purple-leaf *E. ulmoide*, *E. ulmoides*, and Short-branch and dense leaf *E. ulmoide* exerted the greatest antibacterial activities for the different strains. Qinzhong 1–4 showed lower effects of antibacterial activities.

## 7. Chemometrics Analysis of HPLC Fingerprint

### 7.1. Similarity Analysis (SA) of HPLC Fingerprint

Seven varieties of *E. ulmoides* were analyzed to develop a standard fingerprint under the established HPLC conditions (Figure 1b,c). The fingerprinting profiles were generated by a similarity evaluation system for chromatographic fingerprints of TMC (2004 A). Twenty-three and 14 common characteristic peaks were selected from leaf and bark, respectively.

The correlation coefficients of leaf samples ranged from 0.684 to 0.962. The values of the correlation coefficients of bark samples were 0.219–0.973 (Table 7). From Table 3, the leaves of Qinzhong 1–4 (S2, S3, S4, and S5) showed a high degree of similarity. It is suggested that Qinzhong 1–4 had similar chemical constituents. Purple-leaf *E. ulmoides* (S6) was less similar to other varieties. For bark, a high degree of similarity of Qinzhong 1–4 (S2, S3, S4, and S5) was shown. But other varieties showed a low degree of similarity. The samples of different varieties contained slightly diverse chemical constituents. The chromatograms appeared characteristic of the specific components, supporting disparities among the samples.

### 7.2. Hierarchical Clustering Analysis (HCA)

**Figure 2:**
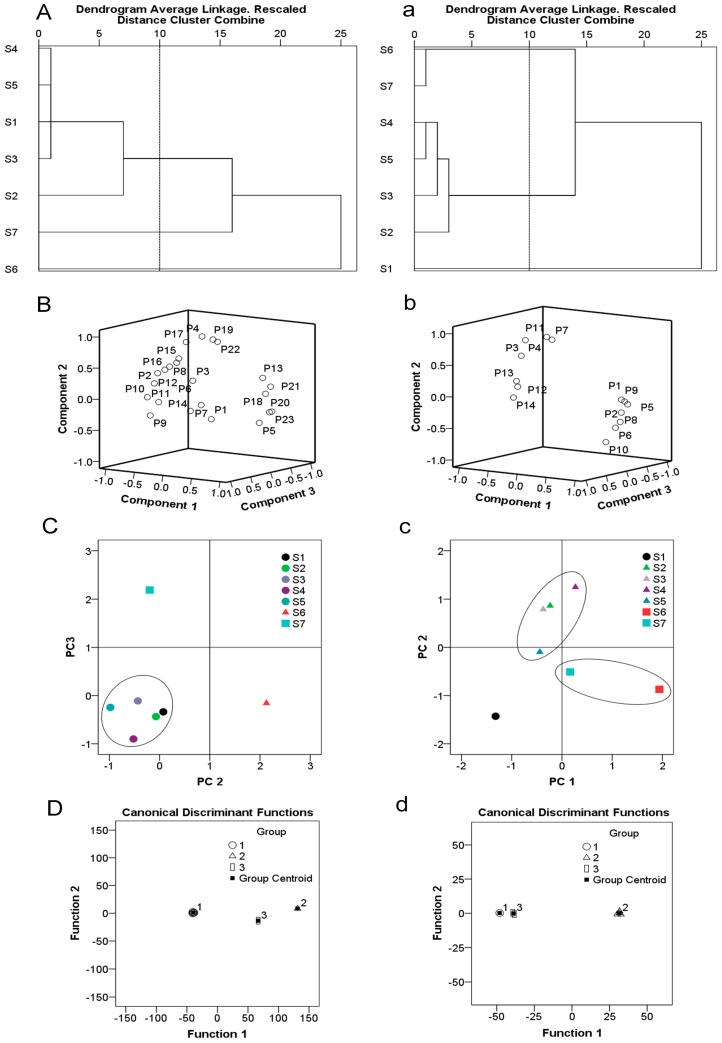
HPLC chemometrics analysis of different *E. ulmoides varieties*. (**A**,**a**): Dendrograms of hierarchical cluster analysis (HCA) for leaf (**A**) and bark (**a**) samples. (**B**,**b**): Loading plot generated from principal component analysis (PCA) for leaf (**B**): peaks 1–23) and bark (**b**): peaks 1–14) samples. (**C**,**c**): Score plot generated from principal component analysis (PCA) for leaf (**C**) and bark (**c**) samples. (**D**,**d**): Canonical discrimination analysis (DA) of HPLC chromatograms for leaf (**D**) and bark (**d**) samples.

HCA assay was selected for quantitative assessment of the seven varieties of *E. ulmoides* and relative peak areas (RPA) of the 23 and 14 common peaks determined for leaf and bark, respectively. A matrix was applied for HCA using SPSS software, and acquired a dendrogram using average linkage between groups and the cosine method. The seven varieties were divided into three groups at a clustering coefficient of 10 both leaf and bark. For the results of leaf, group 1 included S4, S5, S1, S3, and S2 (*E. ulmoides* and Qinzhong 1–4) which were derived from Yangling, Shaanxi province in China. Group 2 contained S7 (Short-branch and dense leaf *E. ulmoides*), while S6 (Purple-leaf *E. ulmoides*) was clustered in group 3, from Ankang, Shaanxi Province, China (Figure 2A). For the results of bark in Figure 2a, it showed group 1 was S1 (*E. ulmoides*). Group 2 included S4, S5, S3, and S2 (Qinzhong 1–4), and group 3 was S6 and S7 (Purple-leaf *E. ulmoides* and Short-branch and dense leaf *E. ulmoides*). From the above, HCA not only could distinguish different varieties, but also differentiated the varieties from their different regions of origin.

### 7.3. Principal Component Analysis (PCA)

To determine the discrimination capacity of the common constituents, PCA was conducted using the RPAs of common peaks, as with HCA as input data. The principal components contained the most information of all variables. For the result of leaf, the first, second, and third PCs were 43.76%, 23.79%, and 15.96%, respectively, accounting for 83.51% of total variability. Thus the three principal components could be assorted for the samples. The results of PCA were consistent with HCA findings, indicative of different chemical profiles from distinct samples. For analysis of the loading plot (Figure 2B) of PC1, PC2 against PC3 revealed that peaks 5, 13, 20, 21, and 23, which contributed to PC1, had an influence on the cluster in a top-down order. By comparison, peaks 4, 17, 19, and 22, mainly contributed to PC2. The score plot of the principal components, PC2 and PC3, visually revealed a positive influence on quality evaluation of the seven varieties of *E. ulmoides* (Figure 2C). For the result of bark, the first three principal components contained the most information of all variables, accounting for 92.28% of total variability. The score plot of the first two principal components, PC1 and PC2, visually revealed a positive influence on quality evaluation of bark samples (Figure 2b,c). Analysis of the loading plots of PC1 against PC2 revealed that peaks explained by peak 1, 2, 5, 6, 8, and 9, had an influence on the cluster in a top-down order. The peaks 4, 7, and 11, mainly contributed to PC2. The scores of the PCA showed the differences in quality among different varieties of *E. ulmoides*. For leaf, the quality of Qinzhong 1 (S2) was the best, followed by Purple-leaf *E. ulmoides* (S6). For bark, Purple-leaf *E. ulmoides* (S6) showed the best quality, followed by Qinzhong 3 (S4). Data from HCA and PCA supported each other and validated the quality evaluation of different varieties of *E. ulmoides* samples.

### 7.4. Discriminant Analysis (DA)

Discriminant analysis was used to build a predictive model to facilitate clustering and distinguishing of group members based on observed characteristics. Based on linear combinations of predictor variables, discriminant function was used to further discriminate and classify the unknown members. The relative peak area of 23 common peaks were selected from leaf samples as variables from the chromatograms; not all the variables generated contributed to the development of the discriminant function. Only valuable variables may be used to generate discriminant functions. Two types of discriminant functions were obtained using SPSS software (IBM SPSS statistics 23, IBM Inc., Chicago, IL, USA).

Canonical discriminant function:

*Y*_1_ = −1.090*X*_4_ + 0.033*X*_14_ − 0.054*X*_16_ + 0.077*X*_17_ − 59.523

*Y*_2_ = 0.183*X*_4_ + 0.002*X*_14_ − 0.187*X*_16_ + 0.004*X*_17_ + 6.391

Fisher’s discrimination function:

*G*_1_ = −22.487*X*_4_ + 0.665*X*_14_ − 0.050*X*_16_ + 1.505*X*_17_ − 224.460

*G*_2_ = −207.204*X*_4_ + 6.308*X*_14_ − 10.719*X*_16_ + 14.602*X*_17_ − 18182.562

*G*_3_ = −140.828*X*_4_ + 4.137*X*_14_ − 3.120*X*_16_ + 9.575*X*_17_ − 8158.537

Standard for discriminant: each sample was assigned to a group according to the highest of the three functional values. G_1_, G_2_, and G_3_ denote samples from groups 1, 2, and 3, respectively, and *X* represents the variable. The variables assigned to the areas of peaks 4, 14, 16, and 17 were adopted to develop the discriminant functions. According to the discriminant standard value obtained, the inputted values of the four variables into the formula were used to classify an unknown sample. The high resolution of DA plots for the three groups were displayed in Figure 2D. The four variables that were employed were the most discriminating, the analytes which were included in groups 1, 2, and 3 could be classified with 100% accuracy.

The relative peak areas of 14 common peaks were selected from bark as variables, and two types of discriminant functions were obtained using SPSS software.

Canonical discriminant function:

*Y*_1_ = 0.016*X*_11_ + 0.248*X*_12_ − 0.023*X*_14_ − 103.319

*Y*_2_ = 0.000*X*_11_ + 0.016*X*_12_ − 0.0.002*X*_14_ − 3.224

Fisher’s discrimination function:

*G*_1_ = 0.898*X*_11_ + 13.755*X*_12_ − 1.275*X*_14_ − 1535.695

*G*_2_ = 2.191*X*_11_ + 33.443*X*_12_ − 3.092*X*_14_ − 9084.553

*G*_3_ = 1.048*X*_11_ + 16.033*X*_12_ − 1.485*X*_14_ − 2085.726

The variables assigned to the areas of peaks 11, 12, and 14 were adopted to develop the discriminant functions. The high resolution of DA plots for the three groups were displayed in Figure 2d. The three variables that employed were most discriminating, the analytes which included in groups 1, 2, and 3 could be classified with 100% accuracy.

According to the HPLC fingerprint results, there is significant variation among the different samples of the seven varieties of *E. ulmoides*. The results of HPLC fingerprint were consistent with SA, HCA, PCA, and DA in our study, and the techniques performed well for the quality assessment*.* For leaf, the quality of Qinzhong 1 was the best and Purple-leaf *E. ulmoides* showed good quality. For bark, Purple-leaf *E. ulmoides* showed the best quality. The results of the quality evaluation of different varieties of *E. ulmoides* were consistent with the results of metabolite profiles and bioactivities. HPLC fingerprint analysis combined with chemometrics methods may be of vital importance to quality evaluation of the seven varieties of *E. ulmoides.* Further studies should include the collection of additional samples from other regions in China, which should be analyzed via HPLC fingerprinting combined with chemometrics, and include the examination of genetic factors.

## 8. Materials and Methods

### 8.1. Plant Materials

Both leaf and bark of the seven varieties of *E. ulmoides* were collected for the study. Collected plant materials were air-dried under shade at room temperature and were stored at −20 °C refrigerator to protect until further analysis.

### 8.2. Preparation of the Extracts

The leaf and bark of seven varieties of *E. ulmoides* (dry weight, 20 g) were separately extracted twice with 400 mL of MeOH (1.5 h) at 70 °C and filtrated. Extracted twice with above method. All extracted solutions were collected and concentrated into the extracts. Extracts were stored at −20 °C in a refrigerator for further analysis.

### 8.3. Total Phenolic, Flavonoid and Gutta-Percha Contents

The total phenolic content was determined using the Folin–Ciocalteau colourimetric method with some modification. The phenolic content was calculated as gallic acid equivalent from the calibration curve of gallic acid standard solutions (20–300 μg/mL) and was expressed as millimole gallic acid equivalent per 100 g of dry weight (mmol equiv. GAE/100 g). The total flavonoid content was determined using the sodium borohydride/chloranil-based (SBC) assay. Samples were prepared at concentration of 0.5 mg/mL. The total flavonoid content of leaf and bark of *E. ulmoides* were expressed as millimole quercetin equivalents per 100 g of dry weight (mmol equiv. QUE/100 g) [30]. Data were reported as mean ± S.D. for three replicates. Significant differences were tested by analysis of variance (ANOVA) by SPSS.

*E. ulmoides* gutta-percha was extracted by high-temperature steaming combined with solvent extraction. The leaf and bark of *E. ulmoides* were separately extracted with deionized water and filtrated. The residue was cooking for 3 h at 150 °C with a solid–liquid ratio of 1:10 of 10% sodium hydroxide. After washing to neutral pH, extraction by ethanol and petroleum ether was conducted in sequence. After freezing centrifugation to obtain gutta-percha, drying, weighing, and calculating the extraction rate was performed [31].

### 8.4. HPLC Analysis of 8 Compounds of Different E. Ulmoides Varieties

Samples were prepared at concentration of 5 mg/mL and then analyzed by RP-HPLC (Agilent Technologies Inc., Santa Clara, CA, USA). The contents of eight compounds (chlorogenic acid, geniposide acid, hyperoside, rutin, astragalin, geniposide, aucubin, and pinealoglycol diglucoside) were quantified using a Agilent Technologies (Santa Clara, CA, USA) 1260 series liquid chromatograph (RP-HPLC) coupled with a variable wavelength detector. The mobile phase consisted of water with 0.2% phosphoric acid (mobile phase A) and acetonitrile (mobile phase B). The following gradient elution program was run: 95% A and 5% B (0 min), 75% A and 25% B (50 min). The detection wavelengths were 208 nm (5 min), 238 nm (8 min), and 254 nm (30 min). The mobile phase flow rate was kept at 0.8 mL/min. The injection volume was 20 μL. Analyses were performed in triplicate.

## 9. Antioxidant Activity

### 9.1. In Vitro Antioxidant Activity (DPPH, ABTS and FRAP)

The antioxidant activity of DPPH was analyzed by the method of scavenging free radicals according to Wang et al. with a few modifications [32]. The results were determined at 517 nm. Trolox was the positive control and a lower IC_50_ value indicated a higher DPPH radical scavenging ability. The ABTS radical scavenging assay was determined by Miller et al. [33]. The results were determined at 734 nm and then were expressed as micromoles of Trolox equiv. per g. The FRAP assay was used to test the ferric reducing ability of the extracts according to Benzie and Strain [34], with some modifications. The absorbance of samples at 593 nm was recorded using the spectrophotometer, and the results were expressed as micromoles of Trolox equivalent per gram. All of the above experiments were repeated in triplicate.

### 9.2. In Vivo Antioxidant Activity (MTS)

*Escherichia coli* were used to determine the antioxidant activity of extracts based on the method described by Smirnova and Zhou [35,36]. The bacteria were incubated in Luria—Bertani (LB) medium at 37 °C with shaking at 120 r/min overnight until they reached the midlog phase. The optical density at 600 nm was used to monitor the cell growth. A quantity of 1 mL of cell suspension was added to 9.0 mL of LB medium to final OD_600_ = 0.25 ± 0.004. Subsequently, 100 μL of samples (100 mg/mL), 1 mL of the diluted cell suspension, and 8.9 mL of LB medium were mixed and incubated at 37 °C with shaking at 180 r/min for 1.5 h. After incubation, 6.5 mM H_2_O_2_ was added, and the absorbance was immediately measured at 600 nm. Incubation was then continued at 37 °C with shaking 180 r/min for 30 min, and the absorbance was measured again. The specific growth rate was calculated according to the following equation:

*μ =* ln(*N*/*N*_0_)/*t*

where *μ* is the specific growth rate, and *N*_0_ and *N* are the optical density at time zero and *t*, respectively. The protective effect of each sample was calculated as follows: the specific growth rate of *E. coli* in medium containing sample and 6.0 mM H_2_O_2_ was divided by the specific growth rate of *E. coli* in medium and H_2_O_2_.

## 10. Antimicrobial Activity (MIC, MBC and EC_50_)

Ten strains were procured from Microbial Culture Collection Center of Guangdong Institute of Microbiology, China. The strains used were *Klebsiella Pneumoniae*, *Escherichia coli*, *Bacillus subtilis*, *Salmonella enteritidis*, *Pseudomonas aeruginosa*, *Staphylococcus aurous*, *Listeria monocytogenes*, *Salmonella paratyphi A*, *Salmonella typhimurium*, and *Candida albicans*.

The antibacterial activity was evaluated by the paper-disc agar diffusion method described by Özer et al. with some modifications [37]. The bacterial suspension was coated on the surface of the Muller–Hinton broth, and the paper-disc (6 mm in diameter) with sample solution was placed on it to calculate the size of the inhibition zone. Streptomycin, tetracycline, and chloramphenicol were used as positive controls and 1% DMSO solution (final concentration) as a negative control. By the result of this procedure, six bacterial species were chosen for further analysis. The MIC and MBC values of the samples were estimated by the agar dilution method [38]. The samples was dissolved in DMSO (10%), and 1.5 mL stock solution incorporated into 13.5 mL Mueller–Hinton broth to obtain concentrations from 3.125 to 100 mg/mL. Then to each plate was added 3 μL bacterial suspensions which were performed in triplicate. The plates were allowed to incubate for 24 h at 37 °C. While the MIC was determined as the lowest concentration of the test samples that showed no visible growth in the culture incubating at 37 °C for 24 h. Then the plates beyond the MIC value continued to be incubated at 37 °C for 24 h. After incubation, the concentration at which no visible growth was seen was determined as the MBC.

The concentration of the samples required for 50% inhibition rate (EC_50_) was calculated using linear regression analysis. The MeOH extract samples of *Eucommia ulmoides* was dissolved in DMSO (10%), and 1.5 mL stock solution was incorporated into 13.5 mL Mueller–Hinton broth to obtain concentrations from 6.25 to 100 mg/mL. Bacterial discs (6 mm in diameter) were placed on the surface of inoculated plates. These plates were incubated at 37 °C for 48 h. Zones of inhibition were measured and recorded. The percentage inhibition was calculated:

[(Diameter_Control_ − Diameter_Test_)/Diameter_Control_] × 100

Tetracycline, chloramphenicol, and streptomycin (5 μg/mL) were used as positive controls, and DMSO (10%) as the negative control. The experiment was repeated thrice and the average values were calculated.

## 11. Conclusions

The quality of seven varieties of *E. ulmoides* were evaluated based on metabolite profiles, bioactivity, and HPLC fingerprint combined with chemometrics analysis. On this basis, the differences of medicinal parts (leaf and bark) were further carried out. For the traditional use of bark, Purple-leaf *E. ulmoides* was suitable. It had the highest contents of total phenolic and gutta-percha, and possessed the strongest antioxidant capacity and antibacterial activities. For the use of leaf, the varieties of Purple-leaf *E. ulmoides* and Qinzhong 1 were befitting. Purple-leaf *E. ulmoides* had the highest bioactivity. Qinzhong 1 had the highest content of chlorogenic acid, aucubin, rutin, hyperoside, and total content of the eight compounds. Combined with SA, HCA, PCA, and DA, the seven varieties of *E. ulmoides* were divided into three groups for the use of leaf and bark. Moreover, the analysis of the HPLC fingerprint coupled with chemometrics analysis not only evaluated quality of the seven varieties of *E. ulmoides*, but also could distinguish different varieties and different regions of origin. Meanwhile, the HPLC fingerprint showed that significant differences in metabolite profiles could exist among these samples.

*E. ulmoides* has great development and commercial potential and provides an important direction for the development and utilization of natural products, which have great advantages for the development of health care products, quality medical products, industrial products, and others. Our results can provide a theoretical basis for *E. ulmoides* resource utilization, qualified evaluation, and cultivation of fine varieties. Because the variety is lower, the next step still need to expand variety selection and geographical scope. This genus of phyto-constituents, as well as pharmacological profile, should be further investigated.

## Figures and Tables

**Table 1:** Contents of total phenolic, total flavonoids, and gutta-percha of leaf and bark of different *E. ulmoides* varieties.

Samples		Total Phenolic Content (mmol equiv. GAE/100 g)	Total Flavonoid Content (mmol equiv. QUE/100 g)	Gutta-Percha Content (%)
Leaf	S1	49.0 ± 2.6 ^c^	256.2 ± 3.4 ^i^	1.81% ± 0.28 ^g^
	S2	13.1 ± 0.1 ^ij^	379.1 ± 2.7 ^c^	2.46% ± 0.09 ^f^
	S3	29.1 ± 0.3 ^e^	366.8 ± 4.4 ^d^	3.33% ± 0.08 ^c^
	S4	9.2 ± 1.6 ^j^	271.3 ± 6.4 ^h^	1.09% ± 0.12 ^h^
	S5	21.8 ± 1.4 ^f^	354.5 ± 2.9 ^e^	1.63% ± 0.25 ^g^
	S6	95.5 ± 3.7 ^a^	288.0 ± 2.6 ^g^	2.96% ± 0.04 ^cd^
	S7	57.4 ± 2.6 ^b^	182.6 ± 1.8 ^l^	1.12% ± 0.11 ^h^
Bark	S1	17.2 ± 1.3 ^ghi^	44.2 ± 4.1 ^m^	4.75% ± 0.05 ^b^
	S2	21.0 ± 0.3 ^fg^	550.4 ± 4.4 ^a^	3.30% ± 0.36 ^d^
	S3	14.4 ± 0.9 ^hi^	398.8 ± 4.6 ^b^	4.80% ± 0.14 ^b^
	S4	15.1 ± 0.2 ^hi^	385.6 ± 6.8 ^c^	2.93% ± 0.06 ^de^
	S5	18.2 ± 1.0 ^fh^	319.2 ± 0.7 ^f^	2.56% ± 0.13 ^ef^
	S6	34.3 ± 0.8 ^d^	230.1 ± 4.1 ^j^	5.46% ± 0.12 ^a^
	S7	14.1 ± 4.8 ^hi^	198.5 ± 2.5 ^k^	2.98% ± 0.02 ^cd^

mmol equiv. GAE/100 g: millimole gallic acid equivalent per 100 g. mmol equiv. QUE/100 g: millimole quercetin equivalent per 100 g. Each value represented in the table are means ± S.D. (*n* = 3). Values with different letters (a, b, c, etc.) within the same column are significantly different (*p* < 0.05).

**Table 2:** Method validation for the quantitative determination of eight compounds.

Compounds	Wavelength (nm)	Regression Equation	Range (μg/mL)	Precision Experiment		Recovery Experiment		Repeatability	
				Area of Peak (mAU × min)	RSD (%)	Average Recovery Rate (%)	RSD (%)	Area of Peak (mAU × min)	RSD (%)
Aucubin	208	y = 1829.7x − 28.307 (R² = 0.9996)	20–2000	221.675 ± 3.46	1.56	100.63 ± 0.13	1.86	1249.4 ± 12.68	1.01
Geniposidic acid	238	y = 6009.2x + 8.9869 (R² = 0.9999)	20–2000	836.875 ± 10.36	1.24	99.29 ± 1.41	1.49	3468 ± 44.85	1.29
Chlorogenic acid	238	y = 7811.3x − 1.014 (R² = 0.9997)	25–3000	1254.48 ± 31.47	2.51	106.32 ± 0.86	1.80	3458 ± 37.32	1.08
Geniposide	238	y = 14552x − 205.23 (R² = 0.9998)	20–2500	8181.1 ± 11.74	0.14	106.43 ± 1.22	1.28	8938.925 ± 27.23	1.49
Pinoresinol Diglucoside	238	y = 2531.7x − 9.934 (R² = 0.9994)	15–2000	713.28 ± 12.58	1.76	103.08 ± 0.77	1.36	3267.5 ± 12.68	1.03
Rutin	254	y = 18116x + 22.911 (R² = 0.9999)	10–1500	1910.93 ± 15.31	0.80	103.33 ± 1.96	1.41	1938.925 ± 27.01	1.39
Hyperoside	254	y = 11847x − 46.079 (R² = 0.9998)	15–2000	1595.15 ± 9.47	0.59	102.14 ± 1.53	1.87	1857.43 ± 35.52	1.91
Astragalin	254	y =20861x + 145.28 (R² = 0.9998)	15–2000	2535 ± 39.05	1.54	96.36 ± 0.98	1.09	2550.7 ± 26.20	1.03

Values were expressed in mean ± S.D. (*n* = 6).

**Table 3:** Contents of the eight compounds of leaf and bark of different *E. ulmoides* varieties (mg/g).

Compounds		Aucubin	Geniposidic Acid	Chlorogenic Acid	Geniposide	Pinoresinol Diglucoside	Rutin	Hyperoside	Astragalin	Total
Leaf	S1	117.2 ± 0.4 ^c^	50.5 ± 1.0 ^c^	60.6 ± 0.4 ^e^	ND	ND	3.0 ± 0.1 ^c^	2.5 ± 0.7 ^f^	0.5 ± 0.1 ^f^	234.3
	S2	91.9 ± 0.1 ^d^	40.6 ± 0.2 ^f^	120.0 ± 0.7 ^a^	2.4 ± 0.1 ^b^	ND	15.1 ± 0.1 ^a^	29.0 ± 0.3 ^a^	3.5 ± 0.1 ^b^	302.5
	S3	85.1 ± 0.8 ^e^	53.0 ± 0.3 ^b^	85.6 ± 0.9 ^b^	1.6 ± 0.1 ^c^	ND	3.9 ± 0.1 ^b^	20.1 ± 0.2 ^b^	2.0 ± 0.1 ^d^	251.3
	S4	65.6 ± 0.8 ^f^	43.1 ± 0.2 ^e^	56.9 ± 0.7 ^f^	1.3 ± 0.1 ^d^	ND	2.7 ± 0.1 ^d^	4.5 ± 0.7 ^e^	0.6 ± 0.0 ^f^	174.7
	S5	36.7 ± 0.6 ^g^	47.3 ± 0.1 ^d^	64.9 ± 0.9 ^d^	2.8 ± 0.1 ^a^	ND	3.9 ± 0.2 ^b^	6.9 ± 0.1 ^d^	1.4 ± 0.1 ^e^	163.9
	S6	121.5 ± 1.4 ^b^	51.3 ± 1.1 ^c^	11.4 ± 0.1 ^g^	ND	ND	4.1 ± 0.1 ^b^	12.2 ± 0.6 ^c^	5.9 ± 0.1 ^a^	206.4
	S7	143.5 ± 0.7 ^a^	117.9 ± 0.1 ^a^	68.5 ± 1.8 ^c^	ND	ND	0.04 ± 0.0 ^e^	4.8 ± 0.2 ^e^	2.5 ± 0.1 ^c^	337.24
Bark	S1	6.6 ± 0.1 ^g^	9.6 ± 0.1 ^f^	2.7 ± 0.4 ^f^	4.5 ± 0.1 ^f^	23.0 ± 1.5 ^f^	ND	ND	ND	46.4
	S2	368.6 ± 1.0 ^a^	87.9 ± 1.7 ^d^	36.5 ± 0.5 ^a^	42.0 ± 0.2 ^c^	44.6 ± 0.4 ^b^	ND	ND	ND	579.6
	S3	210.3 ± 1.0 ^c^	127.1 ± 0.6 ^c^	34.6 ± 0.1 ^b^	58.9 ± 0.1 ^a^	26.5 ± 0.9 ^e^	ND	ND	ND	457.4
	S4	263.8 ± 1.6 ^b^	87.3 ± 0.2 ^d^	22.1 ± 0.5 ^c^	58.6 ± 1.1 ^a^	24.6 ± 0.7 ^ef^	ND	ND	ND	456.4
	S5	196.1 ± 1.7 ^d^	59.2 ± 0.2 ^e^	37.5 ± 0.9 ^a^	52.0 ± 1.0 ^b^	53.6 ± 0.2 ^a^	ND	ND	ND	398.4
	S6	124.8 ± 1.5 ^e^	230.2 ± 1.6 ^a^	4.6 ± 0.1 ^e^	9.1 ± 0.8 ^e^	30.3 ± 1.6 ^d^	ND	ND	ND	399.0
	S7	67.7 ± 0.5 ^f^	137.4 ± 0.2 ^b^	7.2 ± 0.1 ^d^	21.6 ± 0.4 ^d^	40.3 ± 0.3 ^c^	ND	ND	ND	274.2

Each values represented in tables are means ± S.D. (*n* = 3). Values with different letters (a, b, c, etc.) within same column are significantly different (*p* < 0.05). Leaf and bark were calculated to be significantly different separately. ND: not detected.

**Table 4:** Antioxidant activities of leaf and bark of different *E. ulmoides* varieties.

Samples		DPPH IC50 (μg/mL)	ABTS (μmol equiv. Trolox/g)	FRAP (μmol equiv. Trolox/g)	MTS (μ_30__min_)
Leaf	S1	5.1 ± 0.9 ^i^	6527.6 ± 2.0 ^c^	4597.9 ± 0.7 ^c^	1.8 ± 0.2 ^d^
	S2	3.4 ± 0.8 ^k^	3435.7 ± 7.8 ^f^	2471.1 ± 2.9 ^f^	1.7 ± 0.2 ^h^
	S3	2.8 ± 0.6 ^l^	5226.1 ± 2.4 ^d^	3155.4 ± 2.7 ^d^	1.9 ± 0.2 ^g^
	S4	8.4 ± 0.7 ^g^	2820.3 ± 8.3 ^h^	2069.6 ± 0.9 ^g^	1.7 ± 0.1 ^h^
	S5	4.2 ± 0.8 ^j^	4235.1 ± 4.9 ^e^	2697.2 ± 2.89 ^e^	1.8 ± 0.1 ^h^
	S6	1.0 ± 0.7 ^m^	7471.4 ± 2.7 ^a^	6161.6 ± 3.0 ^a^	2.5 ± 0.1 ^a^
	S7	2.8 ± 0.8 ^l^	7324.8 ± 3.8 ^b^	5388.0 ± 1.6 ^b^	2.2 ± 0.2 ^b^
Bark	S1	56.5 ± 0.4 ^a^	1886.6 ± 2.4 ^k^	514.5 ± 2.1 ^n^	1.3 ± 0.1 ^f^
	S2	9.0 ± 0.4 ^e^	1948.3 ± 11.1 ^j^	1548.1 ± 2.1 ^h^	1.6 ± 0.2 ^hi^
	S3	13.1 ± 0.4 ^d^	1103.1 ± 9.1 ^m^	1292.1 ± 2.9 ^j^	1.4 ± 0.1 ^j^
	S4	7.6 ± 0.7 ^h^	902.8 ± 3.8 ^n^	1248.8 ± 3.4 ^k^	1.3 ± 0.1 ^j^
	S5	8.9 ± 0.4 ^f^	1145.7 ± 3.2 ^l^	1518.6 ± 2.1 ^i^	1.6 ± 0.2 ^i^
	S6	28.1 ± 0.2 ^c^	2973.5 ± 2.6 ^g^	713.9 ± 1.1 ^l^	1.9 ± 0.2 ^cd^
	S7	38.0 ± 0.2 ^b^	2202.1 ± 4.3 ^i^	702.3 ± 2.4 ^m^	1.5 ± 0.2 ^e^

The value of Trolox was 16.0 μg/mL. The values represented in the tables are means ± S.D. (*n* = 3). Values with different letters (a, b, c, etc.) within same column are significantly different (*p* < 0.5).

**Table 5:** MIC and MBC values of leaf and bark extracts of different *E. ulmoides* varieties against bacteria.

Samples			Gram Negative				Gram Positive	
			*E. coli*	*P. aeruginosa*	*S. paratyphi*	*S. typhimurium*	*B. subtilis*	*S. aurous*
Leaf	S1 (mg/mL)	MIC	0.3125	0.3125	0.3125	0.6250	0.6250	0.6250
		MBC	2.500	2.500	0.3125	1.250	2.500	0.6250
	S2 (mg/mL)	MIC	0.6250	2.500	2.500	2.500	0.6250	0.6250
		MBC	1.250	5.000	5.000	5.000	1.250	1.250
	S3 (mg/mL)	MIC	1.250	2.500	2.500	2.500	1.250	1.250
		MBC	1.250	2.500	2.500	2.500	2.500	5.000
	S4 (mg/mL)	MIC	10.00	5.000	1.250	1.250	10.00	10.00
		MBC	10.00	5.000	5.000	5.000	10.00	10.00
	S5 (mg/mL)	MIC	1.250	1.250	1.250	1.250	2.500	1.250
		MBC	2.500	2.500	2.500	2.500	5.000	5.000
	S6 (mg/mL)	MIC	1.250	1.250	0.3125	1.250	0.3125	0.3125
		MBC	2.500	1.250	0.3125	1.250	2.500	2.500
	S7 (mg/mL)	MIC	1.250	1.250	1.250	1.250	1.250	0.6250
		MBC	2.500	2.500	2.500	2.500	2.500	2.500
Bark	S1 (mg/mL)	MIC	10.00	10.00	5.000	10.00	0.3125	0.3125
		MBC	10.00	10.00	5.000	10.00	5.000	0.6250
	S2 (mg/mL)	MIC	5.000	2.500	2.500	2.500	5.000	5.000
		MBC	5.000	5.000	5.000	5.000	10.00	10.00
	S3 (mg/mL)	MIC	10.00	5.000	10.00	10.00	5.000	5.000
		MBC	10.00	10.00	10.00	10.00	10.00	10.00
	S4 (mg/mL)	MIC	5.000	10.00	10.00	10.00	10.00	10.00
		MBC	10.00	10.00	10.00	10.00	10.00	10.00
	S5 (mg/mL)	MIC	2.500	10.00	10.00	10.00	2.500	5.000
		MBC	5.000	10.00	10.00	10.00	5.000	10.00
	S6 (mg/mL)	MIC	10.00	2.500	5.000	2.500	0.3125	0.3125
		MBC	10.00	10.00	5.000	10.00	10.00	0.3125
	S7 (mg/mL)	MIC	0.3125	2.500	5.000	0.3125	0.3125	0.3125
		MBC	10.00	10.00	5.000	10.00	5.000	0.3125
Positive Control	Streptomycin (μg/mL)	MIC	5.000	0.3125	1.250	0.3125	0.6250	0.3125
		MBC	5.000	0.3125	1.250	0.6250	0.6250	0.3125
	Tetracyclin (μg/mL)	MIC	1.250	1.250	1.250	2.500	0.6250	1.250
		MBC	5.000	5.000	5.000	10.00	2.500	5.000
	Chloramphenicol (μg/mL)	MIC	2.500	5.000	2.500	5.000	2.500	2.500
		MBC	5.000	5.000	5.000	5.000	2.500	2.500

**Table 6:** EC_50_ values of leaf and bark extracts of different *E. ulmoides* varieties against bacteria and fungi.

Samples		EC_50_ Value (mg/mL)					
		Gram Negative				Gram Positive	Fungi
		*S. enteritidis*	*P. aeruginosa*	*S. paratyphi*	*S. typhimurium*	*S. aurous*	*C. albicans*
Leaf	S1	48.4 ± 0.3 ^b^	19.4 ± 0.2 ^l^	69.3 ± 0.2 ^b^	13.9 ± 0.4 ^o^	40.8 ± 0.3 ^f^	13.2 ± 0.3 ^j^
	S2	33.2 ± 0.2 ^h^	72.2 ± 0.1 ^a^	77.5 ± 0.2 ^a^	67.9 ± 0.4 ^a^	51.7 ± 0.4 ^c^	12.8 ± 0.4 ^j^
	S3	35.9 ± 0.3 ^g^	53.1 ± 0.1 ^c^	59.5 ± 0.3 ^c^	42.1 ± 0.2 ^d^	69.4 ± 0.3 ^a^	79.4 ± 0.3 ^a^
	S4	43.4 ± 0.2 ^d^	35.4 ± 0.2 ^f^	32.7 ± 0.3 ^i^	14.8 ± 0.2 ^m^	37.2 ± 0.4 ^h^	42.2 ± 0.4 ^b^
	S5	37.5 ± 0.2 ^f^	28.5 ± 0.2 ^i^	46.8 ± 0.2 ^f^	23.5 ± 0.3 ^i^	46.3 ± 0.1 ^d^	35.9 ± 0.3 ^c^
	S6	42.5 ± 0.2 ^e^	26.8 ± 0.2 ^j^	14.6 ± 0.1 ^o^	16.2 ± 0.2 ^l^	38.2 ± 0.2 ^g^	16.9 ± 0.2 ^gh^
	S7	46.9 ± 0.3 ^c^	12.9 ± 0.3 ^o^	9.7 ± 0.1 ^p^	33.5 ± 0.3 ^f^	10.6 ± 0.1 ^o^	25.0 ± 0.3 ^e^
Bark	S1	37.9 ± 0.3 ^f^	22.3 ± 0.1 ^k^	27.3 ± 0.2 ^l^	35.4 ± 0.2 ^e^	21.2 ± 0.3 ^j^	14.6 ± 0.3 ^ij^
	S2	21.4 ± 0.2 ^k^	35.4 ± 0.2 ^f^	29.7 ± 0.3 ^k^	51.5 ± 0.3 ^b^	42.4 ± 0.3 ^e^	39.3 ± 0.3 ^c^
	S3	18.4 ± 0.2 ^m^	15.8 ± 0.2 ^n^	57.1 ± 0.2 ^d^	17.0 ± 0.2 ^k^	27.9 ± 0.4 ^i^	40.5 ± 0.2 ^b^
	S4	26.7 ± 0.3 ^j^	56.5 ± 0.2 ^b^	50.0 ± 0.2 ^e^	21.0 ± 0.3 ^j^	37.2 ± 0.4 ^h^	30.6 ± 0.2 ^d^
	S5	32.2 ± 0.2 ^i^	52.1 ± 0.3 ^d^	33.7 ± 0.3 ^h^	23.7 ± 0.4 ^h^	56.5 ± 0.2 ^b^	18.4 ± 0.2 ^g^
	S6	43.9 ± 0.1 ^d^	34.7 ± 0.1^g^	22.9 ± 0.1 ^m^	48.3 ± 0.2 ^c^	20.5 ± 0.2 ^k^	21.4 ± 0.2 ^f^
	S7	52.4 ± 0.4 ^a^	46.8 ± 0.3 ^e^	31.2 ± 0.1 ^j^	33.3 ± 0.2 ^g^	18.3 ± 0.2 ^l^	26.9 ± 0.2 ^e^
Positive Control	Tetracyclin	19.3 ± 0.7 ^l^	17.0 ± 0.4 ^m^	14.8 ± 0.1 ^n^	14.5 ± 0.1 ^n^	15.9 ± 0.3 ^m^	15.9 ± 0.3 ^hj^
	Chloramphenicol	27.3 ± 0.4 ^j^	32.9 ± 0.1 ^h^	34.2 ± 0.1 ^g^	9.4 ± 0.1 ^p^	15.0 ± 0.1 ^n^	9.8 ± 0.2 ^k^

Each of the values represented in the table are means ± S.D. (*n* = 3). Values with different letters (a, b, c, etc.) within same column are significantly different (*p* < 0.05).

**Table 7:** Similarities of chromatograms of leaf and bark of different *E. ulmoides* varieties based on correlation.

Leaf	**No.**	**S1**	**S2**	**S3**	**S4**	**S5**	**S6**	**S7**
	S1	1.000						
	S2	0.775	1.000					
	S3	0.836	0.936	1.000				
	S4	0.962	0.776	0.820	1.000			
	S5	0.924	0.867	0.945	0.927	1.000		
	S6	0.845	0.743	0.759	0.736	0.752	1.000	
	S7	0.945	0.684	0.820	0.877	0.908	0.843	1.000
Bark	**No.**	**S1**	**S2**	**S3**	**S4**	**S5**	**S6**	**S7**
	S1	1.000						
	S2	0.224	1.000					
	S3	0.219	0.916	1.000				
	S4	0.281	0.910	0.936	1.000			
	S5	0.225	0.907	0.922	0.949	1.000		
	S6	0.512	0.538	0.607	0.595	0.482	1.000	
	S7	0.443	0.652	0.729	0.718	0.614	0.973	1.000

## Supplementary Materials

The following are available online, Table S1: The results of recovery experiment, Table S2: Inhibition zone diameter (DIZ) of leaf and bark extracts of different *E. ulmoides* varieties against bacteria and fungi (mm).
