# Supplementary material for: Metabolite Profiles, Bioactivity, and HPLC Fingerprint of Different Varieties of Eucommia ulmoides Oliv.: Towards the Utilization of Medicinal and Commercial Chinese Endemic Tree
Source: Molecules. 2018 Jul 30;23(8):1898. doi: 10.3390/molecules23081898 (PMC6222369; doi:10.3390/molecules23081898)
Supplement: Supplementary file 1 [file molecules-23-01898-s001.pdf]

Table 1. The results of recovery experiment.

Compounds	Contents (mg)			Average recovery rate (%)
	Non-spiked samples	Standards	Spiked samples	
Aucubin	0.543	0.16	0.704	100.63
Geniposidic acid	0.435	0.14	0.574	99.29
Chlorogenic acid	0.245	0.19	0.447	106.32
Geniposide	0.059	0.14	0.208	106.43
Pinoresinol Diglucoside	0.109	0.13	0.243	103.08
Rutin	0.015	0.09	0.108	103.33
Hyperoside	0.018	0.14	0.161	102.14
Astragalin	0.009	0.11	0.115	96.36

Table 2. Inhibition zone diameter (DIZ) of leaf and bark extracts of different *E. ulmoides* varieties against bacteria and fungi (mm).

Samples		Gram negative						Gram positive			Fungi
		K. Pneumoniae	*E.coli*	*S. enteritidis*	*P. aeruginosa*	*S. paratyphi*	*S. typhimurium*	*B. subtilis*	*S. aurous*	*L. monocytogenes*	*C. albicans*
Leaf	S1	7.67±0.76^{defg}	6.83±0.58^{ef}	7.67±0.76^{def}	9.17±2.25^{bc}	6.50±0.50^{g}	11.00±1.74^{c}	12.50±0.50^{c}	7.67±0.58^{cde}	9.67±0.29^{c}	7.17±0.76^{def}
	S2	11.67±0.76^{a}	8.00±1.73^{cde}	9.17±0.29^{de}	6.92±0.14^{de}	6.67±0.58^{fg}	8.17±0.76^{efg}	7.17±0.29^{fg}	7.33±0.58^{def}	6.83±0.29^{fg}	6.67±0.29^{ef}
	S3	8.50±0.87^{bcde}	8.00±1.00^{cde}	8.83±1.04^{def}	6.83±0.29^{de}	7.67±1.15^{def}	9.00±0.50^{def}	7.08±0.14^{fg}	6.17±0.29^{g}	6.83±0.29^{fg}	6.83±0.29^{ef}
	S4	9.83±1.44^{b}	7.08±0.38^{def}	7.17±1.15^{fg}	7.17±0.76^{cde}	8.67±0.76^{cd}	8.00±1.00^{efg}	6.17±0.29^{h}	7.08±0.14^{def}	7.25±0.66^{efg}	6.17±0.29^{f}
	S5	9.00±1.00^{bcd}	8.67±1.04^{cd}	8.33±0.76^{def}	8.33±0.76^{bcde}	6.83±0.29^{efg}	7.33±0.58^{fg}	6.17±0.29^{h}	7.50±0.87^{cdef}	7.17±0.76^{efg}	6.17±0.29^{f}
	S6	7.00±0.50^{efg}	7.17±0.29^{def}	7.42±0.14^{fg}	7.42±0.63^{cde}	9.17±1.26^{bc}	10.67±1.44^{c}	14.33±4.73^{fg}	7.33±0.76^{def}	8.67±1.44^{cd}	7.00±1.00^{ef}
	S7	7.17±1.26^{efg}	9.33±1.04^{c}	7.42±0.95^{fg}	9.67±2.25^{b}	8.17±0.76^{cd}	7.33 ± 1.04^{fg}	12.50±0.50^{c}	7.50±0.50^{cd}	8.00±0.50^{def}	7.00±0.50^{cdef}
Bark	S1	6.17±0.29^{fg}	6.17±0.29^{f}	7.67±0.58^{efg}	7.17±0.76^{de}	7.33±0.29^{efg}	7.33±0.76^{fg}	12.67±3.21^{c}	7.25±0.66^{def}	6.17±0.29^{g}	8.50±1.80^{bc}
	S2	8.83±1.04^{bcd}	8.17±0.29^{cde}	9.33±1.53^{de}	7.58±1.38^{cde}	8.92±0.63^{bc}	6.50±0.00^{g}	6.42±0.38^{gh}	6.83±0.29^{efg}	7.67±1.15^{def}	7.00±0.87^{ef}
	S3	8.83±1.26^{bcde}	9.00±0.87^{c}	11.17±1.89^{c}	8.67±1.53^{bcd}	6.83±0.29^{efg}	8.83±2.02^{g}	7.17±0.76^{fg}	8.00±1.00^{cd}	8.33±0.29^{def}	6.83±0.29^{ef}
	S4	9.67±1.61^{bc}	9.17±1.53^{c}	9.33±0.58^{cd}	6.67±0.29^{e}	7.67±0.58^{def}	10.33±0.58^{def}	7.00±0.00^{ef}	7.17±0.76^{def}	8.33±0.29^{de}	7.67±0.58^{bcde}
	S5	8.50±0.50^{bcde}	6.67±0.29^{ef}	7.50±0.50^{fg}	6.67±0.58^{e}	7.67±0.58^{def}	8.17±1.26^{cd}	7.08±0.14^{fg}	6.83±0.29^{fg}	7.67±1.04^{efg}	8.33±1.15^{bcd}
	S6	6.00±0.00^{g}	6.50±0.50^{ef}	7.42±0.95^{fg}	7.00±0.50^{de}	7.83±0.76^{cde}	6.75±0.43^{efg}	15.50±0.50^{b}	7.83±1.04^{cd}	6.33±0.29^{g}	7.17±0.29^{def}
	S7	6.00±0.00^{g}	6.00±0.00^{f}	6.17±0.29^{g}	6.83±1.04^{e}	7.67±0.76^{def}	7.83±1.89^{efg}	9.83±0.76^{d}	8.00±0.87^{cd}	7.25±1.04^{efg}	6.58±0.14^{ef}
Positive control	Streptomycin	8.00±2.18^{cdef}	24.17±1.26^{a}	19.00±2.65a	17.33±1.04^{a}	13.00±1.32^{a}	18.50±5.50^{efg}	24.17±1.76^{a}	37.00±1.32^{a}	17.67±0.58^{a}	19.00±1.32^{a}
	Tetracyclin	13.67±1.53^{a}	12.33±2.52^{b}	13.33±3.06^{b}	6.50±0.00^{de}	10.50±0.87^{b}	13.00±4.44^{a}	24.17±1.76^{a}	24.83±7.85^{b}	8.67±1.26^{b}	12.00±1.00^{b}
	Chloramphenicol	7.17±0.58^{defg}	7.83±1.53^{cdef}	8.50±0.50^{def}	7.00±0.50^{de}	8.33±1.04^{cdef}	7.67±1.04^{b}	16.3±1.15^{b}	7.08±0.52^{defg}	7.42±0.14^{efg}	9.5±1.50^{def}
Negative control	DMSO	6.00	6.00	6.00	6.00	6.00	6.00	6.00	6.00	6.00	6.00

Each values represented in tables are means ± SD (n = 3). Values with different letters (a, b, c, etc) within same column are significantly different ($p < 0.05$).
